# Utrophin haploinsufficiency does not worsen the functional performance, resistance to eccentric contractions and force production of dystrophic mice

**DOI:** 10.1371/journal.pone.0198408

**Published:** 2018-06-07

**Authors:** Antoine Boulanger Piette, Dounia Hamoudi, Laetitia Marcadet, Frédérique Kyomi Labelle, Rares Ovidiu David, Sabrina Bossé, Anteneh Argaw, Jérôme Frenette

**Affiliations:** 1 Centre Hospitalier Universitaire de Québec, Centre de Recherche du Centre Hospitalier de l’Université Laval (CHUQ-CRCHUL), Université Laval, Quebec City, QC, Canada; 2 Département de Réadaptation, Faculté de Médecine, Université Laval, Quebec City, Canada; University of Sydney, AUSTRALIA

## Abstract

The lack of dystrophin in Duchenne muscular dystrophy (DMD) compromises the integrity and function of muscle fibers. Skeletal muscles, except the diaphragm, do not undergo progressive degeneration in adult mdx mice due to compensatory mechanisms, including structural protein upregulation. New mouse models, including utrophin haploinsufficient mdx (mdx/utrn+/-) mice, may better recapitulate DMD. Our goal was to determine whether mdx/utrn+/- worsens the mdx phenotype and to characterize the course of the disease on muscle function and contractility at 1, 2, and 5 months of age, which encompass all stages of development relevant to DMD therapy. The functional performances of mdx/utrn+/- mice showed that they are not more affected than mdx/utrn+/+ mice based on downhill treadmill running parameters and subsequent recovery measured by open-field voluntary activity. WT mice ran the entire distance (450 m) on the treadmill, with an additional 561 m during the 4 h of open-field while mdx/utrn+/+ and mdx/utrn+/- mice completed, respectively, 236 m and 273 m on the treadmill and 341 m and 287 m during the open-field period. In addition, isolated *ex vivo* contractile properties and repeated eccentric contractions showed that mdx/utrn+/- does not significantly worsen the function of dystrophic EDL muscles, which are mainly composed of fast-twitch fibers that are preferentially affected in DMD. Twitch, absolute tetanic, and specific tetanic forces were very similar in dystrophic EDL muscles from mdx/utrn+/+ and mdx utrn+/- mice at 1, 2, and 5 months of age. Five-month-old mdx/utrn+/+ and mdx/utrn+/- mice lost roughly 50% of their force due to repeated eccentric contractions. Thus, histological, morphological, biochemical functional and contractile observations showed that utrophin haploinsufficiency has a very limited impact on mdx mice.

## Introduction

Duchenne muscular dystrophy (DMD) is a lethal x-linked genetic disease caused by mutations in the dystrophin gene that result in the complete lack of dystrophin protein. The absence of dystrophin in the costameric dystroglycan complex compromises the anchoring of sarcomeres to the extracellular matrix (ECM). In addition, the weak attachment of the cytoskeleton to the sarcolemma and ECM leads to increased susceptibility to contraction-induced damage, particularly eccentric contractions of powerful fast-twitch fibers, causing repeated degeneration-regeneration cycles [1,2]. The loss of sarcolemmal integrity also gives rise to calcium influx, triggering proteolysis and proteasome activation. Necrosis and damage elicit a chronic inflammatory response where contractile tissues are replaced by fibrotic and adipose tissues. Patients eventually lose ambulation, and die due to cardiorespiratory failure. Despite continuous and major efforts, DMD is still treated using an outdated standard of care and represents an unmet need [3].

Several animal models have been developed to investigate novel therapeutic avenues and to decipher the pathophysiology of DMD. The most widely used is the mdx mouse, a genetic model of DMD. The mdx mouse has a point mutation in exon 23 of the dystrophin gene, resulting in a lack of a functional dystrophin protein. Young mdx mice undergo acute phases of degeneration-regeneration shortly after weaning, with muscle necrosis and weakness peaking around 4–6 weeks of age [4–6]. Adult mdx mice appear clinically normal and their limb muscles do not recapitulate the progressive weakness, degeneration and fibrosis of DMD, limiting their use for long term treatment investigations and clinical studies [5,7,8]. Successful regeneration in adult mdx mice is most likely due to multiple compensatory mechanisms, including non-exhaustion of satellite cells, longer telomeres, and upregulation of other structural proteins.

The structural protein utrophin is a partial natural compensatory mechanism in mdx mice and DMD/Becker patients [9–13]. Since utrophin is homologous to dystrophin and since these two proteins may be functionally interchangeable at costamere sites [14], stabilizing the sarcolemma and preventing contraction-induced damage. Utrophin is also found at neuromuscular and myotendinous junctions and in sarcolemmal extrasynaptic regions of regenerating fibers in dystrophic muscles [15–17]. The utrophin levels are correlated with disease severity [10,11] and over expression of this protein seems to improve clinical outcome measures in Becker and DMD patients [15]. In addition, the onset of the disease in mdx mice corresponds to the downregulation of utrophin at the sarcolemma [18]. Interestingly, transgenic [14,19–23] or pharmacological approaches that induce high levels of endogenous utrophin upregulation [24–26] alleviate the dystrophic phenotype in mdx mice and possibly in human patients [27]. Based on the compensatory role of utrophin, dystrophin-utrophin haploinsufficient mdx/utrn+/- and null mdx/utrn-/- mice have been generated to better recapitulate the progressive degenerative processes of DMD [7,28]. Mdx/utrn+/- and null mdx/utrn-/- mice may thus be a useful translational tool for discovering and testing potential therapeutic approaches, particularly for chronic treatments.

Dystrophin-utrophin haploinsufficient mice have slightly shorter lifespans than mdx mice and display a moderate phenotype with select functional tests and histological experiments [29,30]. On the other hand, double-deficient mdx/utrn-/- mice have a lower body weight, widespread muscular fibrosis and necrosis, a reduced lifespan, spinal deformity, joint contractures, and earlier symptoms of cardiomyopathy than their mdx/utrn+/+ and mdx/utrn+/- counterparts [7,28,30–35]. Given the premature death and severity of the symptoms in mdx/utrn-/- mice, mdx/utrn+/- mice may be a good model for evaluating the long-term efficacy of therapeutic interventions. The mdx/utrn+/- has been validated as a potentially more useful model to test anti-inflammatory and anti-fibrotic therapeutic interventions [29,35–37]. However, the extent to which muscle function and physical performance are affected in mdx/utrn+/- mice remain unclear due to the absence of comparative *ex-vivo* muscle contractility data at multiple time-points, and limitations in the analysis of functional assay data [30,37]. Accordingly, the aim of the present study was to characterize the severity and course of the disease in the mdx/utrn+/- mouse model by functional testing using *ex vivo* contractility, morphological, histological and biochemical investigations. We chose the fast-twitch dominant *extensor digitorum longus* (EDL) muscle because fast-twitch fibers are preferentially affected by muscular dystrophy and this muscle is suitable for *ex vivo* contractility experiments. Our findings show that utrophin haploinsufficiency in mdx mice has a limited impact at 1, 2, and 5 months of age in terms of *ex vivo* functional performance, EDL contractile properties and resistance to eccentric contractions.

## Materials and methods

### Animals

Male and female mdx breeding mice heterozygous for utrophin gene (mdx/utrn +/-), derived from the initial dys-/- and utrn -/- models [38,39], were purchased from The Jackson Laboratory. Wild-type mdx/utrn+/+ littermates were used for experiments and background controls. Mice were screened for the desired genotype by polymerase chain reaction (PCR). Wild-type (C57BL/10J; WT) mice were purchased from the Jackson Laboratory and were bred at our specific pathogen-free (SPF) animal facility. Food and water were provided ad libitum. At the end of the different experimental procedures, the mice were euthanized under anesthesia by cervical dislocation. All procedures were approved by the Université Laval Research Center Animal Care and Use Committee based on Canadian Council on Animal Care guidelines.

### Genotyping

Genomic DNA was isolated from mouse tail tissue samples, and a fragment of the utrophin gene was amplified by PCR according to Jackson Laboratory genotyping protocol Standard PCR: Utrn^tm/Ked^alternate1(https://www2.jax.org/protocolsdb/f?p=116:5:0::NO:5:P5_MASTER_PROTOCOL_ID,P5_JRS_CODE:24119,014563). Mutant forward (5’-CGCTTCCTCGTGCTTTACGGTAT-3’), WT forward (5’-TGTCATTCTCTGAGGCCTTTC-3’) and common reverse (5’-AAGATTTGCAGACCGGAAGA-3’) primer sets were used to screen for mutant and WT utrophin alleles. The thermocycler conditions were as follows: 95°C for 30 s, and forty 30-s cycles at 93°C, 53°C for 1 min, and 68°C for 1 min, followed by 68°C for 5 min with Hot Start TAQ Polymerase (New England Biolabs). Genotyping data is presented in S1 Fig.

### Treadmill exercise

Five-month-old wild-type (WT), mdx/utrn +/+, and mdx/utrn +/- mice were acclimatized with three sessions on a motorized treadmill at 6, 8, and 10 m/min for 5 min. On the day of the experiment, the mice ran downhill for 45 min on a motorized treadmill with a 14-degree slope at 10 m/min. They were observed continuously during the running protocol. Exhausted mice showing physical signs of discomfort were rested for 1 min before resuming the protocol. The running bout was discontinued after 3 stops. Following the 45-min downhill protocol, voluntary activity was measured using video tracking software in a 50 x 50 cm open field for 4 h [40]. One- to two-month-old mice are not recommended for the downhill treadmill test as they are generally unable to perform this stressful exercise.

### Inverted grid grip duration test

This test was conducted using a 50 x 30-cm metallic framework with a 1-cm square grid and a 1.5-mm rod diameter placed 20 cm above a cushioned surface. Two separate sessions were used to acclimatize 1-, 2- and 5-month-old mice. On the day of the protocol, the mice subjected to the test until they could no longer hold on to the grid. The mice were continually observed to ensure that grip failure was due to exhaustion. In the case of voluntary abandonment, the test was repeated 1 h later. Data are expressed as holding impulse, a measure of sustained force: body weight (9.806 x 10^−3^ N/gram) x grip duration.

### Grip strength test

The four-limb grip strength analysis was performed using a grip meter (Columbus Instruments). One-, 2-, and 5-month-old mice were placed on a grid that they grasped, before being pulled backwards at a controlled speed and angle until they could no longer hold on to the grid. The test was repeated three times on three separate days, and the average of the maximal values was used to estimate strength normalized by body weight.

### Isometric contractile properties

The mice were weighed, injected with buprenorphine (i.p. 0.1 mg/kg), and anesthetized with pentobarbital sodium (i.p. 50 mg/kg) 15 min later. EDL muscles were then carefully resected and were attached to an electrode and a force sensor (305B-LR dual-mode, Aurora Scientific Inc.) controlled by Dynamic Muscle Control Analysis unit and data acquisition software (Aurora Scientific Inc.). Unlike the diaphragm (DIA) muscle, EDL muscles are not progressively impaired in mdx adult mice, making them suitable for use as a more severe model of muscular dystrophy. During the contractile property experiments, the muscles were incubated at 25°C in oxygenated Krebs-Ringer solution supplemented with 2 mg/mL of glucose [41,42]. Once the optimal length (L0) had been determined, the muscles were stimulated for 500 ms at 10, 20, 50, 80, 100, and 120 Hz to induce subtetanic and tetanic contractions and to determine the force-frequency curves. Twitch tension (Pt, g) and maximum tetanic tension (P0, g) values were recorded and were analyzed using Dynamic Muscle Data Analysis software (Aurora Scientific Inc.). Muscle resistance to eccentric contractions was evaluated using 7 contractions at 150 Hz for 700 ms with a 1-min rest period between contractions. The eccentric contraction protocol consisted of 500-ms tetanic contractions at 150 Hz followed by muscle lengthening at 0.5 L0/s for 200 ms. Force values were calculated as the percentage of the initial force produced. The length of the muscles was measured, the tendons were removed, and the muscles were weighed in order to calculate their specific force using the following formula: sP0 (N/cm2) = P0 (g) x Lf (mm) x muscle density (mg/mm^3^)/muscle mass (mg), where Lf is L0 x 0.44 and muscle density is 1.062. Contralateral EDL muscles were embedded in OCT compound, frozen in isopentane cooled in liquid nitrogen, and stored at −80°C until used for the histological assays.

### Histological assays

Transverse sections of EDL muscles (10 μm) were cut mid-belly (Leica Microsystems CM1850). Hematoxylin and eosin (H&E) staining (Sigma-Aldrich) was used to assess the cross-sectional areas (CSA) of the fibers as well as central nucleation. Five images of transversal sections per muscle were acquired at 200x magnification, which represents approximately 300 myofibers per muscle. To quantify the area of fibrotic aggregation, five sections per muscle were stained with Masson’s Trichrome and fibrosis was identified at 200x magnification. The sections were examined using an inverted microscope (Nikon, Canada) and were analyzed using ImageJ software version 1.49i (NIH, Bethesda, MD).

### Collagen content

EDL muscles were weighed, rapidly frozen in liquid nitrogen and stored at −80°C. Collagen content was determined using a hydroxyproline assay adapted from Treat-NMD SOP DMD_M.1.2.006. Briefly, muscles were dried overnight at 56°C, weighed, and hydrolyzed for 3h in 0.2 ml of 6N hydrochloric acid at 130°C. Hydrolysates were mixed with 1 mL of distilled water 100 μL red methyl and 150 μL 2,5N NaOH for neutralization. Next, 200 μL of hydrolysate were mixed with 100 μl of 0,05M chloramine-T in citrate buffer and oxidized at RT for 20 min. The samples were then mixed with 100 μl of perchloric acid for 5 min. Finally, 20% p-dimethylaminobenzaldehyde was added and incubated for 45 min at 60°C. Quantification was determined by extinction measurement of the resulting solution at 560 nm. A standard curve 0–6,000 μM L-hydroxyproline (Sigma) was included in each assay. Results are reported in micrograms of hydroxyproline per milligrams of wet tissue weight.

### Creatine kinase activity

Serum creatine kinase (CK) activity was quantified using the enzyme-coupled assay reagent from Fisher Scientific (Pointe Scientific Creatine Kinase CK10), according to manufacturer’s instructions and the procedure from Treat NMD_M.2.2.001. Serum samples were analyzed in duplicate using an infinite F200 plate reader (TECAN), Data are expressed as units per liter (U/L).

### Western blot for model validation

Snap-frozen EDL muscles were homogenized in RIPA lysis buffer containing protease inhibitor cocktail (Sigma-Aldrich). 30 ug of protein homogenates were electrophoretically separated on 4/15% gels (Bio-Rad) and transferred to polyvinylidene difluoride membranes (Bio-Rad), blocked in 5% skim milk, and incubated overnight at 4°C with anti-utrophin primary antibody 1/500 (Santa Cruz Biotechnology). The membranes were washed and incubated with horseradish peroxidase-conjugated secondary antibody (Santa Cruz Biotechnology). Protein bands were revealed using the ECL-Plus chemiluminescent detection system (Perkin-Elmer) and were normalized for loading using Gelcode Blue Stain reagent (Thermo Scientific). ImageQuant LAS 4000 biomolecular imager was used to detect a chemiluminescent signal and analyzed using Quantity One software (version 4.6.6; Bio-Rad). Representative utrophin blot is presented in S1 Fig.

### Statistical analyses

All values are expressed as means ± SEM. The data were analyzed using a one-way ANOVA, or a two-way ANOVA for repeated eccentric contractions, followed by Tukey’s test (InStat Graphpad). The levels of significance were set at * p<0.05, ** p<0.01, and *** p<0.001 for comparisons between WT and mdx/utrn+/+ or mdx/utrn+/- mice. The level of significance was set at § p<0.05 for comparisons between mdx/utrn+/+ and mdx/utrn+/- mice.

## Results

### Mdx/utrn+/- and mdx/utrn+/+ mice display similar functional performances

**Fig 1:**
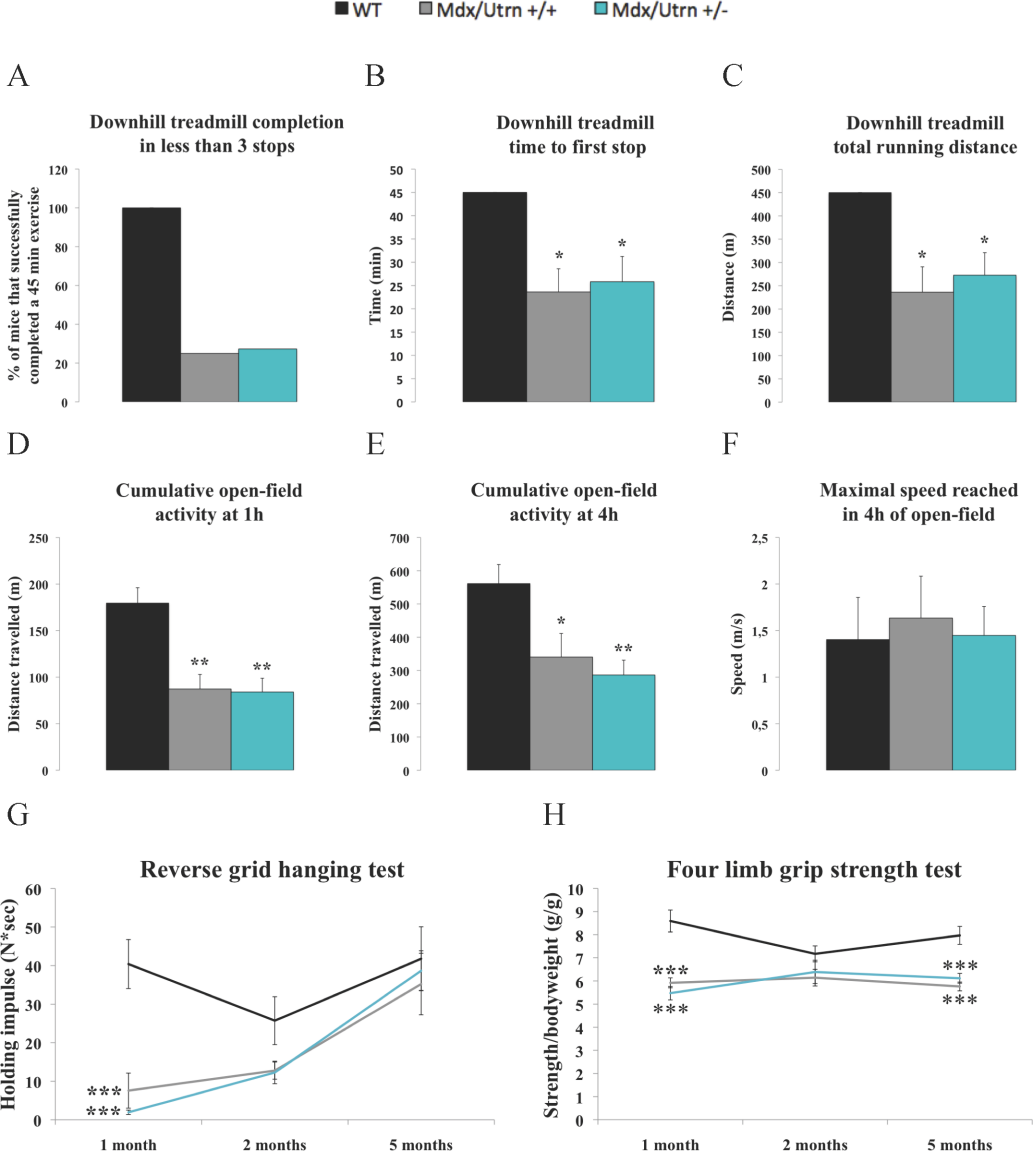
Functional performances of wild-type, mdx/utrn +/+, and mdx/utrn +/- mice. Five-month-old mdx/utrn+/+ and mdx/utrn+/- mice have a significantly lower treadmill completion rate (A), time to first stop (B), total running distance (C), distance travelled at 1 h post-treadmill exercise (D,) and distance travelled at 4 h post-treadmill exercise (E) than WT mice. The maximum speed reached by the three experimental groups was not significantly different during the 4 h open-field observation period (F). Hanging test performance was significantly lower in 1-month-old mdx/utrn+/+ and mdx/utrn+/- mice (G). Four limb grip strength performance was also significantly lower in 1- and 5-month-old mdx/utrn+/+ and mdx/utrn+/- (H). Overall, utrophin haploinsufficiency in mdx mice did not induce a time-related deterioration of functional tasks relative to mdx/utrn+/+ mice. Data are expressed as means ± SEM. * p<0.05, ** p<0.01, and *** p<0.001 for comparisons between WT and mdx/utrn+/+ or mdx/utrn+/- mice; § p<0.05 for comparisons between mdx/utrn+/+ and mdx/utrn+/- mice; analysis of variance using Tukey’s post hoc test. n = 6–9.

To assess functional resistance to eccentric contractions and the associated post-stressful exercise short-term voluntary activity, 5-month-old mice were tested on a treadmill with a 14-degree decline at 10 m/min for 45 min. Once the treadmill exercise over, mice voluntary activity was monitored in an open-field using a video tracking system. The treadmill completion rate of mdx/utrn+/+ (25%) and mdx/utrn+/- (28%) mice was significantly lower than that of WT mice (100%), (Fig 1A). The time to first stop was 23.6 min for the mdx/utrn+/+ mice (*p*<0.05) and 25.8 min for the mdx/utrn+/- mice (*p*<0.05) while the WT mice never stopped (Fig 1B). The total running distance was proportionally lower for the mdx/utrn+/+ mice (236 m) (*p*<0.05) and the mdx/utrn+/- mice (273 m) (*p*<0.05) compared to WT mice (450 m, i.e., the maximum distance) (Fig 1C). For the open-field activity, the distances travelled 1 h and 4 h post-treadmill exercise were significantly lower for the mdx/utrn+/+ mice (87 and 340 m, respectively, *p*<0.05) and the mdx/utrn+/- mice (84 and 287 m, respectively, *p*<0.05) than for the WT mice (180 and 561 m, respectively) (Fig 1D and 1E). There were no differences between the groups in terms of the maximum speed reached, an indicator of power, during the 4 h open-field observation (mdx/utrn+/+: 1.6 m/s; mdx/utrn+/-: 1.5 m/s; and WT: 1.4 m/s) (Fig 1F). The inverted grid grip test was used to characterize muscular endurance. The grip test performances of the 1-month-old mdx/utrn+/+ (7.6 N*s, *p*<0.05) and mdx/utrn+/- (1.9 N*s, *p*<0.05) mice were significantly lower than that of the WT mice (40.4 N*s) (Fig 1G). Since the 5-month-old mdx/utrn+/+ and mdx/utrn+/- mice weighed significantly more than the WT mice, we normalized grip strength to body weight (*p*<0.05) (Table 1). The four limb grip strength performances of the 1-month old mdx/utrn+/+ (5.9 g/g, *p*<0.05) and mdx/utrn+/- (5.5 g/g, *p*<0.05) mice were significantly lower than that of the WT mice (8.6 g/g) (Fig 1H). The four limb grip strength performances of the 5-month-old mdx/utrn+/+ (5.8 g/g, *p*<0.05) and mdx/utrn+/- (6.1 g/g, *p*<0.05) mice were also significantly lower than that of the WT mice (8.0 g/g) (Fig 1H). Overall, utrophin haploinsufficiency in mdx mice did not induce a more severe time-related deterioration of functional tasks relative to mdx/utrn+/+ mice.

**Table 1:** Body weight and morphological characteristics of EDL muscles from wild-type, mdx/utrn +/+, and mdx/utrn +/- mice at 1, 2, and 5 months of age.

	1 Month			2 Months			5 Months		
	WT	Mdx/ Utrn+/+	Mdx/ Utrn+/-	WT	Mdx/ Utrn+/+	Mdx/ Utrn+/-	WT	Mdx/ Utrn+/+	Mdx/ Utrn+/-
**Body** **weight** **(g)**	18,63 ± 0,32	19,25 ± 0,53	18,38 ± 1,07	24,54 ± 0,67	25,88 ± 0,64	26,63 ± 0,65	31,00 ± 0,78	34,21 ± 0,79 **	34,00 ± 0,49 *
**EDL mass** **(mg)**	6,39 ± 0,19	6,99 ± 0,31	7,69 ± 0,42 *	9,87 ± 0,30	11,36 ± 0,58	12,20 ± 0,81 *	9,78 ± 0,42	15,58 ± 0,93 ***	18,51 ± 0,95 *** §
**EDL** **mass/ body** **weight**	0,34 ± 0,01	0,36 ± 0,01	0,42 ± 0,01 ***§	0,41 ± 0,01	0,44 ± 0,02	0,45 ± 0,02	0,32 ± 0,02	0,45 ± 0,02 ***	0,54 ± 0,03*** §
**EDL** **L0** **(mm)**	10,66 ± 0,24	10,17 ± 0,13	10,16 ± 0,18	12,34 ± 0,29	11,92 ± 0,34	11,79 ± 0,41	12,68 ± 0,24	13,31 ± 0,15	13,15 ± 0,10

EDL muscle mass is significantly higher in 1- and 2-month-old mdx/utrn+/- mice than in WT mice. The EDL mass/bodyweight ratio of 1-month-old mdx/utrn+/- mice is significantly higher than the ratios of 1-month-old mdx/utrn+/+ and WT mice. Five-month-old mdx/utrn+/+ and mdx/utrn+/- mice have significantly higher bodyweights, EDL masses, and EDL mass/bodyweight ratios than WT mice. Five-month-old mdx/utrn+/- mice have a significantly higher EDL mass and mass/bodyweight ratio than mdx/utrn+/+ mice. Data are expressed as means ± SEM.

* p<0.05

** p<0.01

*** p<0.001 for comparisons between WT and mdx/utrn+/+ or mdx/utrn+/- mice

§ p<0.05 for comparisons between mdx/utrn+/+ and mdx/utrn+/- mice; analysis of variance, with Tukey’s post hoc test. n = 6–14.

### Utrophin haploinsufficiency has a mild effect on *ex vivo* EDL muscle contractility and has no impact on resistance to eccentric contractions

**Fig 2:**
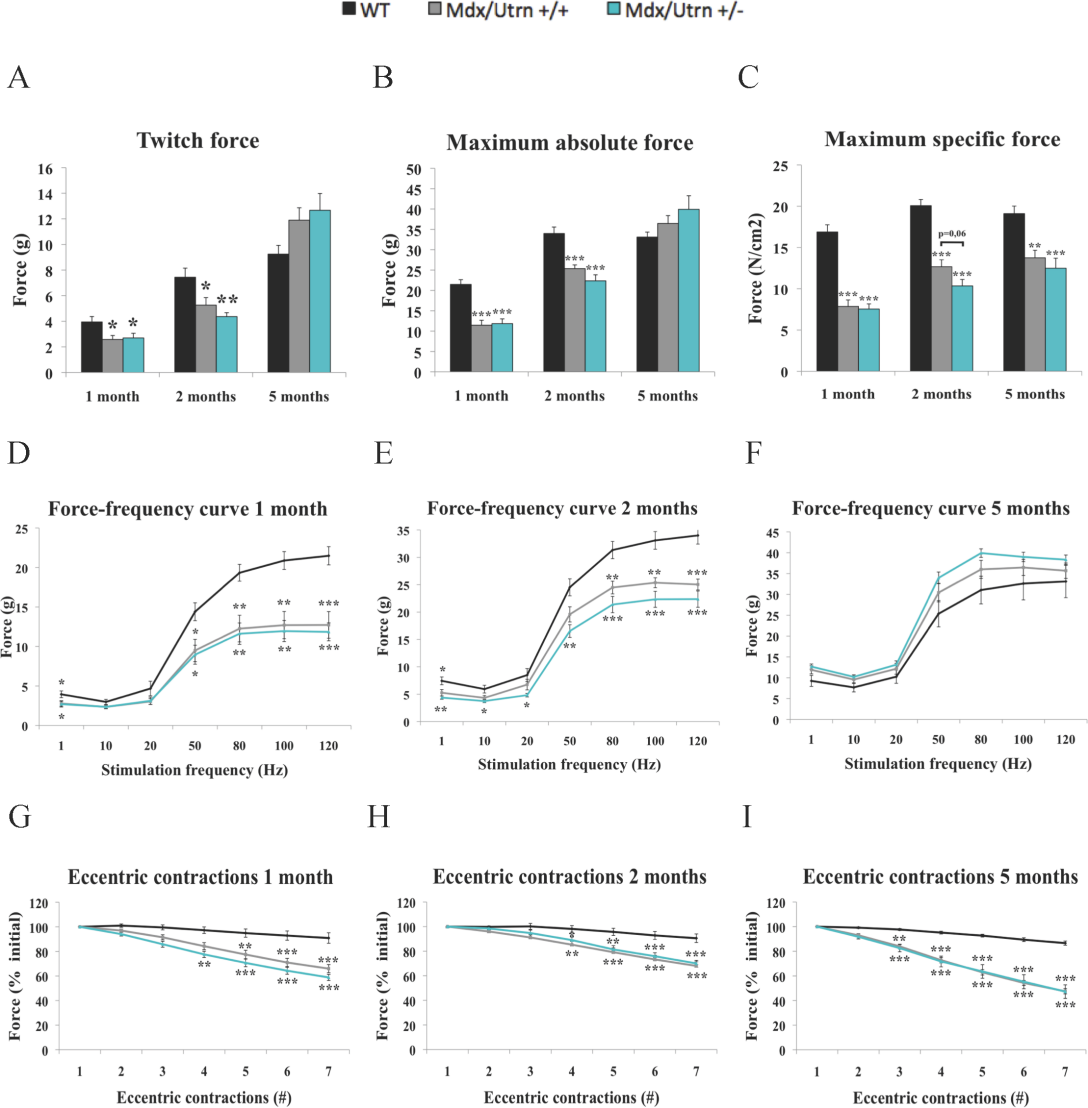
*Ex vivo* contractile properties and resistance to seven eccentric contractions of extensor digitorum longus muscles from wild-type, mdx/utrn +/+, and mdx/utrn+/- mice. The twitch (A) and absolute tetanic (B) forces of EDL muscles are significantly lower at 1 and 2 months of age in mdx/utrn+/+ and mdx utrn+/- mice compared to WT mice. In addition, the maximum specific force is significantly lower at 1, 2, and 5 months of age in mdx/utrn+/+ and mdx/utrn+/- mice compared to WT mice. A tendency for the maximum specific force to be lower in mdx/utrn+/- mice than in mdx/utrn+/+ mice can be seen at 2 months of age (p = 0.06). The force-frequency curves of the EDL muscles show that there is a marked decrease in force production at 1 month of age in mdx/utrn+/+ and mdx utrn+/- mice, which is in agreement with the maximum absolute force results (D). The force frequency curves show that there is a progressive recovery of dystrophic EDL muscles at 2 months of age (E) and a full recovery at 5 months of age in mdx/utrn+/+ and mdx utrn+/- mice (F). All dystrophic muscles at all ages are vulnerable to *ex vivo* eccentric contractions (G, H, I). The contractile properties and the eccentric contraction results show that utrophin haploinsufficiency does not worsen the function of fast-twitch dystrophic EDL muscles, which is consistent with the functional performance results. Data are expressed as means ± SEM. * p<0.05, ** p<0.01, and *** p<0.001 for comparisons between WT and mdx/utrn+/+ or mdx/utrn+/- mice; § p<0.05 for comparisons between mdx/utrn+/+ and mdx/utrn+/- mice; analysis of variance with Tukey’s post hoc test. n = 6–12.

To further investigate the effect of utrophin haploinsufficiency, we assessed the *ex vivo* contractile properties, the gold standard for testing isolated skeletal muscle function, of the three groups of mice. The twitch (Fig 2A) and absolute tetanic (Fig 2B) forces of the EDL muscles of the mdx/utrn+/+ mice (35–47% and 29–26% decrement, respectively) and the mdx utrn+/- mice (32–43% and 41–35% decrement, respectively) were significantly lower than those of the WT type mice at 1 and 2 months of age (*p*<0.05). The twitch and absolute tetanic forces of dystrophic EDL muscles of mdx/utrn+/+ mice and mdx utrn+/- mice at 1, 2, and 5 months of age were very similar (Fig 2A and 2B). In addition, the maximum specific forces of 1-, 2-, and 5-month-old mdx/utrn+/+ and mdx/utrn+/- mice were significantly lower than that of WT mice (*p*<0.05). The maximum specific force of 2-month-old mdx/utrn+/- mice (10.3 N/cm^2^) was lower than that of 2-month-old mdx/utrn+/+ mice (12.7 N/cm^2^) and this difference was not statistically significant (Fig 2C). The force-frequency curves of EDL muscles from 1-month-old mdx/utrn+/+ and mdx utrn+/- mice were much lower than that of WT mice, indicating a marked decrease in force production (Fig 2D). However, the force-frequency curves also indicated that the force production of dystrophic EDL muscles is partially recovered at 2 months of age (Fig 2E) and is fully recovered at 5 months of age in the two dystrophic mouse strains (Fig 2F). Dystrophic muscles are vulnerable to *ex vivo* eccentric contractions, which account for as much as 50% of the loss of force in 5-month-old mdx/utrn+/+ and mdx/utrn+/- mice (Fig 2G–2I). Therefore, the levels of utrophin expression in the mdx/utrn+/- mice seem sufficient to prevent any additional loss of force following repetitive eccentric contractions.

### Utrophin haploinsufficiency has limited effects on selected histological, morphological and biochemical features of dystrophic EDL muscles

**Fig 3:**
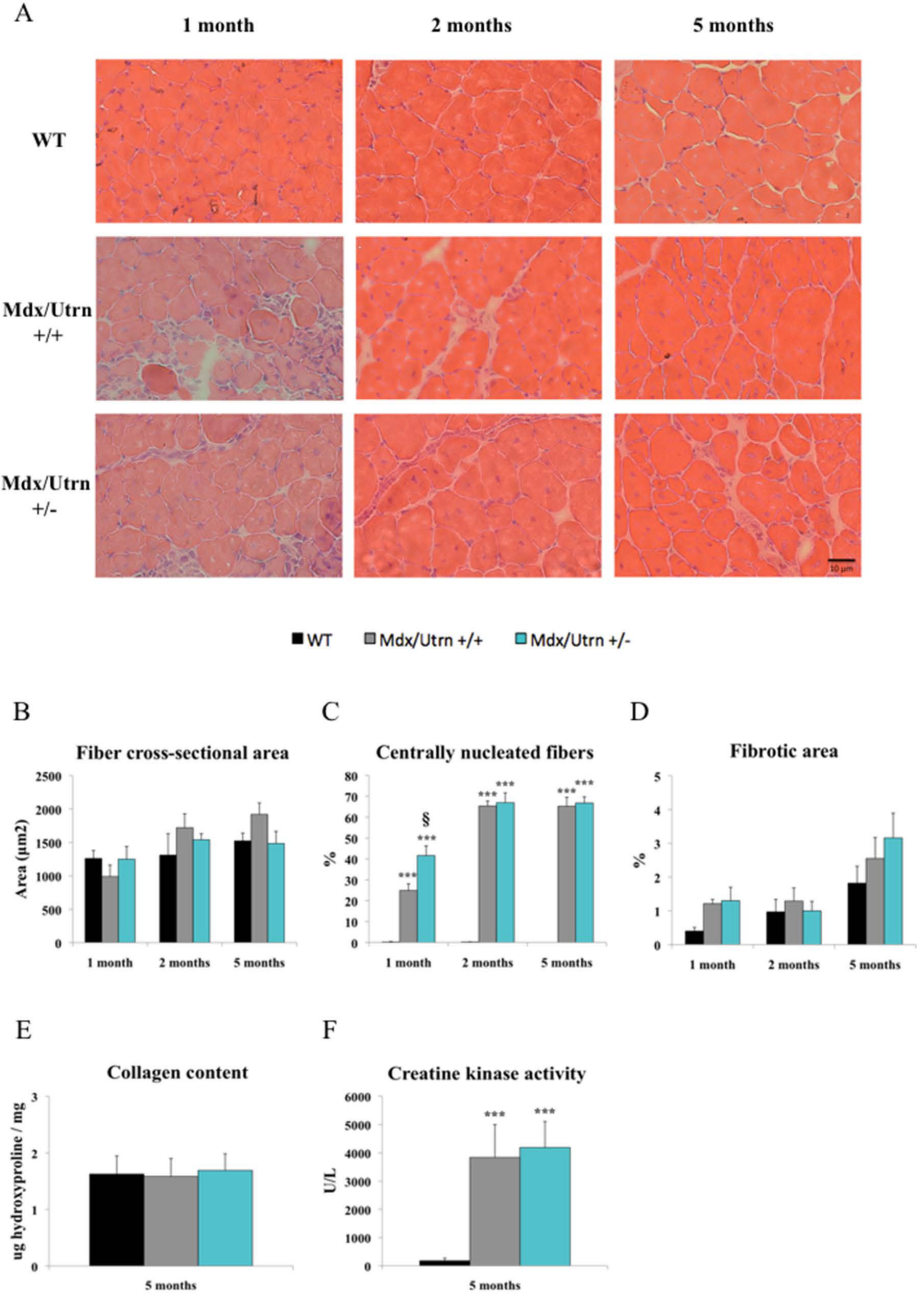
Histological, morphological and biochemical features of wild-type, mdx/utrn+/+, and mdx/utrn+/- mice. H&E staining of EDL muscle sections from WT, mdx/utrn +/+, and mdx/utrn +/- mice at 1, 2, and 5 months of age (A). Fiber cross-sectional area measurements of EDL muscles show no significant fiber hypertrophy in mdx/utrn+/+ and mdx/utrn+/- mice compared to WT mice (B) while the number of centrally nucleated fibers is significantly higher in mdx/utrn +/- mice at 1 month of age (C). Non-significant changes in the extent of fibrosis can be seen in any of the muscles tested at all ages using the fibrotic area calculation (D) or the whole muscle collagen content based on hydroxyproline content measurements at 5 months (E). Non-significant differences in the activity of creatine kinase were found between dystrophic mice at 5 months (F). Data are expressed as means ± SEM. * p<0.05, ** p<0.01, and *** p<0.001 for comparisons between WT and mdx/utrn+/+ or mdx/utrn+/- mice; § p<0.05 for comparisons between mdx/utrn+/+ and mdx/utrn+/- mice; analysis of variance with Tukey’s post hoc test. n = 4–9.

We next analyzed the effect of utrophin haploinsufficiency on the histological and morphological features of dystrophic EDL muscles. The CSAs of myofibers from 1-, 2-, and 5-month-old mdx/utrn+/+ and mdx/utrn+/- mice were the same, and were not significantly different from those of WT mice (Fig 3B). We then assessed the number of centrally nucleated myofibers as an index of muscle degeneration and regeneration. When compared to WT mice, the number of centro-nucleated myofibers increased by 24% in mdx/utrn+/+ mice and by 40% in mdx/utrn+/- mice. While the number of centro-nucleated fibers in mdx/utrn+/- mice was significantly different from the number in mdx/utrn+/+ mice at 1 month of age (*p*<0.05), no differences were seen at 2 and 5 months (Fig 3C). Furthermore, there were no differences in the fibrotic areas in the three groups of mice at 5 months of age (Fig 3D), as confirmed by whole-muscle collagen content determined using the hydroxyproline assay (Fig 3E). Finally, we quantified serum CK level as an indirect marker of muscle damage [43]. At 5 months, CK concentration increased equally but very significantly by respectively 21,5 and 23,5-fold in mdx/utrn+/+ and mdx/utrn+/- relative to WT mice (Fig 3F).

## Discussion

The development of new therapeutic approaches for DMD requires robust and reliable animal models, aiming at improving muscle integrity and resistance to eccentric contraction-induced muscle damage. Mdx/utrn+/- mice have been proposed as a more severe and representative model of DMD. However, there is currently no consensus on the severity and underlying *ex vivo* and *in vivo* muscle functions in mdx/utrn+/- mice. Extensive functional, contractile, and histomorphological analyses are thus required to confirm the superiority of this dystrophic mouse model.

The downhill running protocol induces severe muscle damage and is a reliable approach for measuring the vulnerability of dystrophic muscles [44]. We showed that downhill running performance is severely impaired and post-treadmill activity is markedly reduced in both mdx/utrn+/+ and mdx/utrn+/- mice, which is consistent with the structural role assigned to dystrophin [1]. These results validate the use of the downhill treadmill protocol for adult mdx mouse studies. In addition, the voluntary activities of the mdx/utrn+/+, mdx/utrn+/-, and WT mice that did not perform the running protocol was similar (data not shown), ruling out inherent variations that may influence the outcome. The functional performances observed in the open-field assay paralleled the reverse grid holding impulse test and the four-limb grip strength test in the mdx/utrn+/+ and mdx/utrn+/- mice. These results were also in agreement with the normalized forelimb grip strength values reported in the literature for 1- to 4-month-old mdx/utrn+/+ and mdx/utrn+/- mice [30]. Nonetheless, conflicting results have been reported in other specific experimental conditions. For example, the grip duration of mdx/utrn+/- mice (1 to 3 months of age) has been reported to be shorter than that of mdx/utrn+/+ mice [37], while the time spent hanging during the two and four limbs hanging tests have been reported to be shorter with mdx/utrn+/- mice (1 to 4 months of age) [30]. These discrepancies could possibly be due to differences in body weight between the two strains. In the present study, the normalized holding impulse and grip strength values for the mdx/utrn+/+ and mdx/utrn+/- mice at certain ages were the same as those for the WT mice, questioning the sensitivity and precision of these tests for genotype-phenotype evaluations, particularly in these two mouse models where skeletal muscles are not equally affected and compensatory mechanisms may take place throughout disease evolution [1]. Nevertheless, our results suggest that the level of utrophin in skeletal muscles from mdx/utrn+/- mice is sufficient to generate comparable functional performances and to provide similar resistance to eccentric contractions to mdx/utrn+/+ mice.

To further investigate potential differences in muscle functions between mdx/utrn+/+ and mdx/utrn+/- mice, we studied *ex vivo* contractile properties of fast-twitch EDL muscles. Utrophin haploinsufficiency had a very limited impact on a number of contractile parameters at all tested ages. At 1 month of age, the mdx/utrn+/+ and mdx/utrn+/- mice produced the same levels of twitch, absolute and specific maximal forces, with similar resistance to repeated eccentric contractions, all of which were, as expected, largely inferior to the values for WT mice. The differences between WT, mdx/utrn+/+, and mdx/utrn+/- mice in terms of absolute and specific tetanic force production were not surprising since it is well documented that mdx mice undergo the most important and severe peak of muscle degeneration immediately after weaning. However, utrophin haploinsufficiency in mdx mice did not exacerbate the loss of force following eccentric contractions. Muscular dystrophy in terms of twitch and absolute maximum forces and functional performances in both phenotypes partially subsided at 2 months of age. However, the maximal specific force of mdx/utrn+/- mice tended to be slightly and transiently lower than that of mdx/utrn+/+ mice (p = 0.06) at 2 months of age, and the gain in muscle mass was highly effective for maintaining absolute force at 5 months of age in both mdx/utrn+/+ and mdx/utrn+/- mice [45]. A previous study showed that mice doubly-deficient in dystrophin and utrophin are very vulnerable to eccentric contractions [46]. Resistance to *ex vivo* eccentric contractions was significantly impaired in dystrophic mice. EDL muscles from mdx/utrn+/- and mdx/utrn+/+ mice were equally vulnerable to lengthening contractions clearly demonstrating the similarity between the two dystrophic mice.

To further confirm our functional and contractile results, we performed histological experiments. We found that the average CSA of myofibers, which include hypertrophied and small degenerating/regenerating myofibers, remain relatively stable in the WT, mdx/utrn+/+, and mdx/utrn+/- mice. The number of centrally nucleated myofibers was significantly higher in the EDL muscles of 1-month-old mdx/utrn+/- mice than in the EDL muscles of mdx/utrn+/+ mice, suggesting that mdx/utrn+/- mice may have a more profound and earlier cycle of degeneration-regeneration. This histological difference did not affect EDL contractile properties. We also observed a similar level of fibrosis in 1-, 2-, and 5-month-old mdx/utrn+/-, mdx/utrn+/+, and WT mice, suggesting that the gain in muscle mass of dystrophic EDL muscles is most likely associated with edema and fat cell accumulation. There is a lack of consensus on fibrosis in limb skeletal muscles. For example, fibrosis in the gastrocnemius of mdx/utrn+/- mice has been reported to be higher at 2 months of age but not at 10 months of age. On the other hand, no fibrosis has been observed in the quadriceps of mdx/utrn+/-mice at 3 or 4 months of age but an increase has been observed at 6 months of age [29,30,35]. Furthermore, no differences in fibrotic/necrotic tissue has been observed in the tibialis anterior or triceps of mdx/utrn+/- mice at 4 months of age [30]. Overall, the loss of contractile function seems to occur earlier than histological changes in dystrophic mice.

### Conclusion

Our findings that utrophin haploinsufficiency does not worsen the functional performance of mdx mice and the force production of EDL muscles suggest that the monoallelic expression level of utrophin is sufficient in mdx/utrn+/- mice to prevent further deterioration of the muscle. Accordingly, previous works have shown that low level expression of dystrophin in the severe mdx/utrn-/- mouse model protects dystrophic muscles and may provide a vital benefit [47,48]. Mdx mice remain a cost-efficient model for drug discovery in particular for testing *ex vivo* contractile properties and resistance to eccentric contractions, specifically during the first peak of muscle degeneration at 4-5-weeks of age, or following downhill eccentric contraction protocol during adulthood. Overall our results indicate that EDL muscles are equally compromised between 1 and 5 months of age in mdx and mdx/utrn+/- mice.

## Supporting information

S1 Fig Representative genotyping data and utrophin protein level in EDL muscles of wild type, mdx/utrn+/+, mdx/utrn+/- and mdx/utrn-/- mice. PCR-based genotyping confirmation of utrophin alleles in mdx/utrn+/+, mdx/utrn+/- mice (A). Western blotting shows that utrophin protein level is increased in EDL muscles from mdx/utrn+/+ and mdx/utrn+/- compared to WT, and decreased in mdx/utrn+/- compared to mdx/utrn+/+ (B). As expected, utrophin is absent in mdx/utrn-/- EDL muscles.
(TIFF)

## Abbreviations

CSA: cross-sectional area
DIA: diaphragm
DMD: Duchenne muscular dystrophy
EDL: *extensor digitorum longus*
GM: *gastrocnemius*
L0: optimal length
P0: maximum absolute force
PCR: polymerase chain reaction
Pt: twitch force
sP0: maximum specific force
TA: *tibialis anterior*
WT: wild-type
