# Supplementary figures and images for: Utrophin haploinsufficiency does not worsen the functional performance, resistance to eccentric contractions and force production of dystrophic mice
Source: PLoS One. 2018 Jun 7;13(6):e0198408. doi: 10.1371/journal.pone.0198408 (PMC5991729; doi:10.1371/journal.pone.0198408)

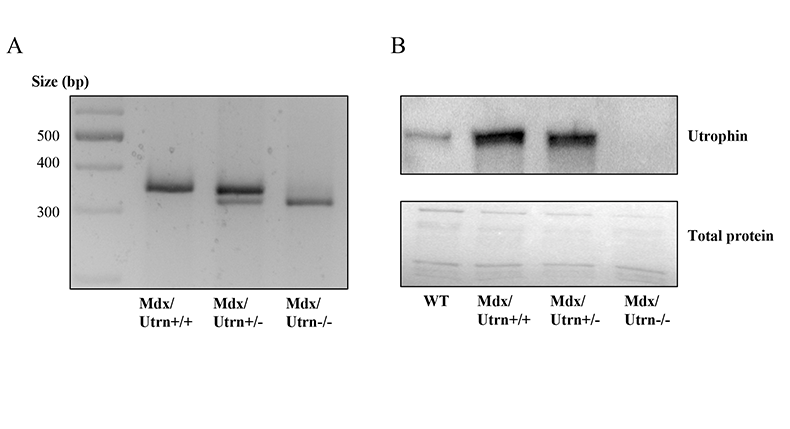

Supplement: S1 Fig — PCR-based genotyping confirmation of utrophin alleles in mdx/utrn+/+, mdx/utrn+/- mice (A). Western blotting shows that utrophin protein level is increased in EDL muscles from mdx/utrn+/+ and mdx/utrn+/- compared to WT, and decreased in mdx/utrn+/- compared to mdx/utrn+/+ (B). As expected, utrophin is absent in mdx/utrn-/- EDL muscles. (TIFF) [file pone.0198408.s001.tiff]
